# Destructive arthritis in a patient with chikungunya virus infection with persistent specific IgM antibodies

**DOI:** 10.1186/1471-2334-9-200

**Published:** 2009-12-10

**Authors:** Denis Malvy, Khaled Ezzedine, Maria Mamani-Matsuda, Brigitte Autran, Hugues Tolou, Marie-Catherine Receveur, Thierry Pistone, Jérome Rambert, Daniel Moynet, Djavad Mossalayi

**Affiliations:** 1 Travel Clinics and Tropical Disease Unit, Department of Internal Medicine, Infectious Diseases and Tropical Medicine, University Hospital Center, Bordeaux, F-33075 France; 2 Centre René Labusquière (Tropical Medicine and Hygiene Branch), University Bordeaux, Bordeaux, F-33076, France; 3 PPF Parasitology, University Bordeaux 2, Bordeaux, F-33076, France; 4 Laboratory of Immunity and Infection, UMR-S INSERM 945, University Pierre and Marie Curie, Hôpital Pitié-Salpêtrière, 83, Boulevard de l'hôpital, Paris F-75651, France; 5 Laboratory of Virology, French Armed Forces Institute of Tropical Medicine (IMTSSA), Allée du Médecin colonel Eugène Jamot, Le Pharo, BP 60109, 13262 Marseille Cedex 07, France

## Abstract

**Background:**

Chikungunya fever is an emerging arboviral disease characterized by an algo-eruptive syndrome, inflammatory polyarthralgias, or tenosynovitis that can last for months to years. Up to now, the pathophysiology of the chronic stage is poorly understood.

**Case presentation:**

We report the first case of CHIKV infection with chronic associated rheumatism in a patient who developed progressive erosive arthritis with expression of inflammatory mediators and persistence of specific IgM antibodies over 24 months following infection.

**Conclusions:**

Understanding the specific features of chikungunya virus as well as how the virus interacts with its host are essential for the prevention, treatment or cure of chikungunya disease.

## Background

Chikungunya virus (CHIKV) is an enveloped positive-strand RNA alphavirus which can infect human epithelial and endothelial cells, fibroblasts or macrophages [1,2]. A CHIKV epidemic recently occurred on islands of the Indian Ocean in 2005 [3-5]. CHIKV infection may cause an algoeruptive syndrome with disabling joint pain and recurrent rheumatic manifestations [6-8]. Until now, it has been assumed that complete recovery occurs, even when symptoms are long lasting. Remarkably, despite the severity and duration of arthritis, articular destruction has been reported very rarely [9]. We report the case of a patient with CHIKV infection presenting with severe chronic rheumatism accompanied by progressive destructive arthritis and dysregulated expression of inflammatory mediators.

## Case presentation

In November 2005, a 60-year-old French man living in La Réunion experienced an acute influenza-like illness with diffuse arthralgia affecting bilaterally the distal inter-phalangeal joints of the fingers and the toes with hand tenosynovitis. His past medical history was unremarkable with no family history of inflammatory rheumatism.

Serology demonstrated the presence of anti-CHIKV IgM and confirmed the diagnosis of CHIKV infection. During the following months, the patient had persisting inflammatory arthralgia and joint stiffness which were not improved by symptomatic treatment. One year later, he developed refractory tenosynovitis in the wrists.

On February 15, 2007, the patient returned to France and consulted in our department. He complained of persistent symmetrical inflammatory arthritis of the wrists with fixed oedema of the two hands predominating on the right. Hand synovitis of the extensors and the flexors of wrists and fingers were noted. Lymphocyte immunophenotyping showed an increased CD4 T-cell count at 1,18 × 10^9^/L (63.5%) and an activated CD45/CD3 (-) T-cell count at 0.209 × 10^9^/L (11.3%), and CD45/CD3 (+) at 0,119 × 10^9^/L (6.4%). Serum immunoglobulin was normal, as were the C3 and C4 complement fractions. No markers of autoimmunity were found, notably anti-citrullin peptide antibodies, antinuclear antibodies or cryoglobulinemia. The HLA B27 gene was positive and HLA system class II genotyping revealed an HLA-DRB1.03.11 genotype.

**Figure 1:**
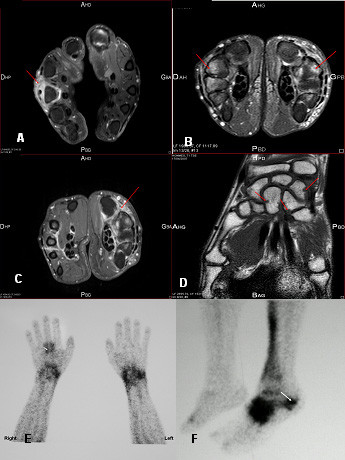
**Magnetic resonance imaging (MRI) and bone scintigraphy of the hands and wrists of a 60-year-old man with chikungunya virus infection revealing**. A. Arthritis of the 3^rd ^metacarpo-phalangeal joint of the right hand with extensor tenosynovitis associated with intra-articular swelling (red arrow on axial section, time-resolved contrast-enhanced T1-weighted sequence after Gadolinium injection with fat suppression) B. Bilateral periostum inflammation and oedematous carpitis with synovitis predominating on the left hand (arrow on axial section, time-resolved-enhanced T2-weighted sequence with fat suppression) C. Asymmetric inflammatory carpitis with multiple synovitis of flexors of the left wrist (red arrows on axial section, time-resolved contrast-enhanced T1-weighted sequence after Gadolinium injection with fat suppression) D. Bone destruction on the left hand (red arrows on coronal section, time-resolved contrast-enhanced T1-weighted sequence with fat suppression) E. Bone scintigraphy of the wrists and hands showing an intense focus of technetium 99 m-labeled methylene disphosphonate tracer uptake, particularly in the right metacarpo-phalangeal wrist. F. Bone scintigraphy of the left foot showing an intense focus of technetium 99 m-labeled methylene disphosphonate tracer uptake, particularly in the left ankle.

At the time of the consultation, serologic status for CHIKV antibodies was reevaluated using IgM-capture and an IgG-capture enzyme-linked immunoabsorbent assay with inactivated cell-culture-ground chikungunya virus and mouse anti-chikungunya hyperimmune ascitic fluid (Institut Pasteur, Lyon, France). Persistent specific anti-CHIKV IgM was detected in this late stage serum sample, collected 18 months after the infection, with optical density (OD) values of 1.47 for IgG and 0.81 for IgM. Testing for CHIKV RNA was negative [10]. Radiography of the hands and wrists showed a subchondral defect of the 2^nd ^and 3^rd ^right proximal interphalangeal finger joints as well as of the 3^rd^, 4^th ^and 5^th ^left distal interphalangeal joints. Magnetic resonance imaging (MRI) of the hands and wrists revealed marked bilateral periostal inflammation and oedematous carpitis (Fig 1A and 1B), with carpis synovitis (1C) and bone destruction in the left hand (1D) accompanied by intra-articular swelling (1D). Bone scintigraphy revealed diffuse inflammation of several joints, prominent in the right wrist (3^rd ^metacarpo-phalangeal joint) (Fig 1E) and the left ankle (1F), as well as evolutive enthesopathy of the left calcaneum. Methotrexate (MTX) was initiated at the dose of 17.5 mg/week and four months later, dramatic improvement was observed in both the number and state of swollen and tender joints and in tendon involvement. At this time, MRI of the hands, wrists and feet showed reduced progression of erosion and a decrease in radiographic inflammation and oedematous damage compared to before treatment. Clinical and radiological improvement was maintained over 15 months. At this end-point, CHIKV antibody serology showed persistence of both specific IgM and IgG, with OD values of 0.60 and 0.32, respectively.

### Immunological evaluation

T cell function was assessed using proliferation assays and ELISPOT quantification of interferon-γ (IFN-γ) production by T cells. No proliferative response or IFN-γ production could be detected in response to CHIKV antigens derived from an in-house preparation of β-propionolactone-inactivated virus (French Armed Forces Institute of Tropical Medicine, Marseilles, France). T cell viability was ascertained by concurrent detection in the same assays of T cells responding to candidin and herpes simplex virus-1 antigens tested as controls.

Peripheral blood lymphocytes (PBL) from the patient and from three healthy control individuals were isolated and analysed at a transcriptomic level using real-time reverse transcriptase-polymerase chain reaction (PCR) to detect mRNA species encoding inflammatory mediators (RT^2 ^Profiler™ PCR Array Human Inflammatory Cytokine and Receptors, SuperArray, Frederick, USA). Each PCR used the same quantity of total RNA from each cell culture and the absence of a signal after 35 PCR cycles was taken as the threshold for the mRNA not being detectable. These PCR analyses were completed by measurement of cytokine levels in the serum and in the 48 h PBL-derived supernatant. These analyses were performed on samples obtained before treatment initiation and at the four-month follow-up.

At baseline, characteristic up regulation of mRNAs encoding pro-inflammatory mediators, including TNF-α, LTA, CRP, IL-1A and IL-17C was observed in PBL from the patient (Table 1). In addition, these cells expressed high levels of mRNAs encoding chemokines (IL-8, CCL1, CCL7, CCL8, CCL16, CCL24, CCL26, and CXCL5) or their receptors (XCR1, CCR2, CCR6, CCR8, and CCR9). In contrast, the expression of genes encoding CD40 Ligand (CD40L), IFN-α, IL-22, CXCL2 and CXCL14 was not detected. High levels of the pro-inflammatory cytokines IL-1β, IL-6, IL-8 and IL-10 were detected in the supernatant following 48 h incubation of PBLs in culture medium alone, while comparatively low levels of IFN-γ and TNF-α were found. On the contrary, IFN-α, IL-4 or IL-5 were not detected in cell supernatant (Table 2).

**Table 1:** Gene expression profile in a patient with chikungunya virus infection before and four months following methotrexate (MTX) treatment.

Gene Name	Unigene ID	Description	Before MTX Fold/Control	After MTX Fold/Control	Before/after Fold ††
***Inflammatory mediators***					

**TNF-α**	241570	Tumor necrosis factor-α (TNFsuperfamily, member 2)	9†	-15	-135

**LTA**	36	Lymphotoxin alpha (TNF superfamily, member 1)	1	-10	-13

**CRP**	76452	C-reactive protein, pentraxin-related	12	-3	-33

**IL-1A**	1722	Interleukin 1, alpha	2	-5	-9

**IL-1F5**	516301	Interleukin 1 family, member 5 (delta)	84	1	-90

**IL-1F6**	278910	Interleukin 1 family, member 6 (epsilon)	2	-9	-20

**IL-1F7**	166371	Interleukin 1 family, member 7 (zeta)	4	-3	-11

**IL-1F8**	278909	Interleukin 1 family, member 8 (eta)	7	-4	-28

**IL-1F9**	211238	Interleukin 1 family, member 9	8	-2	-20

**IL-1RN**	81134	Interleukin 1 receptor antagonist	49	-3	-167

**IL-9**	960	Interleukin 9	3	-6	-20

**IL-9R**	406228	Interleukin 9 receptor	5	-3	-15

**IL-17C**	278911	Interleukin 17C	10	1	-10

**SPP1**	313	Secreted phosphoprotein 1 (osteopontin, bone sialoprotein I, early T-lymphocyte activation 1)	7	-2	-15

**C3**	529053	Complement component 3	12.25	19	1

***Th2-related factors***					

**IL-13**	845	Interleukin 13	5	-11	-55

**IL-22**	287369	Interleukin 22	**OFF**	**OFF**	--

**CD40L**	592244	CD40 ligand (TNF superfamily, hyper-IgM syndrome)	**OFF**	**OFF**	--

***Inflammatory chemokines and their receptors***					

**IFN-α**	211575	Interferon-α2	**OFF**	**OFF**	--

**IL-8**	624	Interleukin 8	5	1	-5

**XCR1**	248116	Chemokine (C motif) receptor 1	34	-2	-62

**CCL1**	72918	Chemokine (C-C motif) ligand 1	4	-31	-109

**CCL7**	251526	Chemokine (C-C motif) ligand 7	3	-14	-40

**CCL8**	271387	Chemokine (C-C motif) ligand 8	2	-46	-80

**CCL16**	10458	Chemokine (C-C motif) ligand 16	6	-3	-22

**CCL24**	247838	Chemokine (C-C motif) ligand 24	76	-3	-190

**CCL26**	131342	Chemokine (C-C motif) ligand 26	7	-2	-15

**CCR2**	644637	Chemokine (C-C motif) receptor 2	13	89	7

**CCR6**	46468	Chemokine (C-C motif) receptor 6	4	-5	-17

**CCR8**	113222	Chemokine (C-C motif) receptor 8	8	-2	-19

**CCR9**	225946	Chemokine (C-C motif) receptor 9	5	-3	-15

**CXCL2**	**590921**	**Chemokine (C-X-C motif) ligand 2**	**OFF**	**2**	**ON**

**CXCL5**	89714	Chemokine (C-X-C motif) ligand 5	27	4	-7

**CXCL14**	**483444**	**Chemokine (C-X-C motif) ligand 14**	**OFF**	**1**	**ON**

Patient had persistent specific IgM antibodies and erosive arthritis for two years before treatment.

† gene expression in patient peripheral blood mononuclear cells compared to those observed in cells from three healthy controls using real-time RT-PCR. Results shown as fold expression compared to control values. Inflammatory arrays were performed using RT2 Profiler PCR Array System (Ref: APHS-011B, SuperArray, Frederick, USA, http://www.superarray.com

†† Comparison of gene expression before MTX treatment and following 4 months therapy

**Table 2:** Cytokine profiles from patient peripheral blood mononuclear cell supernatants before and four months following methotrexate (MTX) treatment.

	**pg/ml**^†^		
	
		Patient cells^††^	
Cytokine	Healthy controls	*Before MTX*	*After MTX*
IFN-γ	0-10	13	<10

IL-1β	0-10	1 100	2

IL-6	0-35	9 482	864

IL-8	150-2000	1 4795	1094

IL-10	0-10	425	4

TNF-α	0-60	92	<10

^† ^Cytokine levels in supernatants from cells incubated 48 h in medium alone and quantified using FlowCytomix Human Th1/Th2 Kit as recommended (Bender Medsystems, Vienna, Austria, http://www.bendermedsystems.com).

^†† ^No cytokine was detected in serum. IFN-α, IL-2, IL-4, IL-5 and IL-12 were not detected in these samples.

Four months following initiation of treatment, the expression of most of the above mRNAs was attenuated, and expression of CD40L, IFN-α and IL-22 remained undetectable. At this time, cytokine levels measured in the cell culture supernatant were markedly decreased and surface CD40L expression on peripheral blood cells could not be detected (data not shown).

## Conclusions

Chikungunya virus infection is usually a self-limiting disease characterized by arthralgia with late peripheral joint pain in the smaller joints [11,12]. A sole case of late articular destruction after decades of post CHIKV-chronic rheumatism has been reported with a questionable relationship to the CHIKV infection [9]. Our case is remarkable for the clearly erosive arthritis pattern accompanied with enthesopathies, which developed within less than two years of the index CHIKV infection [13].

The patient was positive for HLA B27 antigen although no clinical rheumatologic manifestations had been noted before the CHIKV infection. For this reason, we can conclude that he developed a form of spondylarthropathy. Concerning the acute phase, recent findings suggested that both classical and severe symptoms of chikungunya disease closely reflect CHIKV tissue tropism [14], with fibroblasts being the primary targets, as they are for other alphaviruses [1,15]. The molecular basis of this tropism may combine specific interactions between virus and host cells and tissues, and an intrinsic lower ability of this cell type to control CHIKV infection [8,14].

The pathogenesis of persistent arthritis remains unclear although persistent infection of synovial macrophages has been documented for other alphaviruses [16,17]. Moreover, factors secreted by infected macrophages seem to play a pivotal role in joint inflammation [18]. To date, the presence of CHIKV RNA in joints during the acute phase of infection has only been demonstrated in animal models [14,19]. CHIKV RNA has never been detected in joint tissues during the late phase, although it has been found in human muscle satellite cells three months after infection [2]. Taken together with reports of the presence of long lasting IgM anti-CHIKV antibodies [20], these findings could indicate the persistence of CHIKV in certain host tissues or cells. However, there is no evidence that viral persistence is necessary to sustain chronic joint pathology after the infection. In our case, an antigen-specific immunopathology may underlie the development of chronic joint disease due to the presentation of specific autologous or microbial arthritogenic peptides [21]. This would be consistent with the notion of an infectious trigger for immune activation derived from molecular mimicry which has been postulated as a potential pathogenic mechanism in autoimmunity [22-24].

High levels of pro-inflammatory cytokines IL-1, IL-6 and IL-8 were detected in cell supernatants from the patient's lymphocytes and elevated expression of genes encoding a variety of cytokines and chemokines known to be implicated in the pathogenesis of arthritis were also observed [25,26]. In contrast, IFN-α, IL-4 and IL-5 were not detected in cell supernatants whereas IL-10 was increased. Of interest, the lymphocytes failed to express genes encoding IFN-α, IL-22 and CD40L, which are required for B cell differentiation, optimal immunoglobulin isotype switching and antiviral activity. Although the persistence of specific IgM antibodies has been documented after CHIKV infections [20], an association between these antibodies and disease severity has not been established. It is possible that antibody persistence may be determined by the persistence of virus in host tissues since, in acute arboviral infections, IgM are generally no longer detectable after 6-12 months. Unfortunately, in our case, it was not possible to perform RT-PCR for detection of CHIKV in synovial fluid or tissue

Viruses have evolved a number of mechanisms for suppressing host immune responses. In particular, viral proteins may suppress the retinoic acid-inducible gene I-like helicase and Toll-like receptor pathways important for the expression of type 1-IFNs, such as IFN-α isoforms, which play a crucial role in eliminating viral infections [14,27-29]. Immunoglobulin class switching from IgM to IgG and IgA is central to immunity against viruses and requires the activation of B-cells by T-cells via cytokine interactions with CD154/CD40L. Mutations in the CD40L gene have been observed in cases of inherited hyper-IgM X-linked immunodeficiences [27]. In this context, CHIKV may be able to evade T-cell dependent IgG and IgA responses by attenuating CD40L gene expression. Such a mechanism has been postulated previously to explain hyper IgM syndrome associated with various viral infections [30-32].

We report here a case of CHIKV infection with joint and bone erosion and persistent specific IgM nearly two years following infection. Transcriptomic and protein analysis revealed individual genes which could be implicated in the pathogenesis of chronic arthritis; these genes have not been associated previously with alphavirus-induced arthritis.

## Consent

The patient has provided written consent to the use of his history for publication

## Competing interests

The authors declare that they have no competing interests.

## Authors' contributions

DMa took care of the patient. DMa and KE drafted the manuscript. BA and HT performed immunoglobulin assays. MMM, DMoy, DMa, JR and DMos performed the immunological analyses and evaluation. All authors reviewed the manuscript, contributed to its finalisation and approved the submitted version.  

## Pre-publication history

The pre-publication history for this paper can be accessed here:

http://www.biomedcentral.com/1471-2334/9/200/prepub
